# Síndrome del nodo sinusal enfermo ¿marcapaso o ablación? Reporte de caso

**DOI:** 10.47487/apcyccv.v4i4.324

**Published:** 2023-12-27

**Authors:** Elvihots Yorddy Alberto Ayauja López, Ángel Cueva-Parra, Hael Fernández-Prado, Gerald Lévano-Pachas

**Affiliations:** 1 Unidad de Electrofisiología, Servicio de Cardiología, Hospital Nacional Guillermo Almenara Irigoyen, Lima, Perú. Unidad de Electrofisiología Servicio de Cardiología Hospital Nacional Guillermo Almenara Irigoyen Lima Perú

**Keywords:** Síndrome del Nodo Enfermo, Síncope, Vía Accesoria Atrio-ventricular, Ablación con Catéter, Sick Sinus Syndrome, Syncope, Accessory Atrioventricular Bundle, Catheter Ablation

## Abstract

El síndrome taquicardia-bradicardia es la forma de presentación más frecuente del síndrome del nodo sinusal enfermo, y comúnmente se caracteriza por episodios de fibrilación auricular paroxística seguidos de pausas significativas, sobre todo en pacientes adultos mayores; otras taquiarritmias frecuentemente asociadas son la taquicardia auricular y el *flutter* auricular. La asociación entre una taquicardia ortodrómica y pausas significativas en estos pacientes es una presentación poco habitual. Presentamos el caso de un adulto mayor con síndrome taquicardia-bradicardia asociado a síncope, que presentaba episodios de taquicardia incesante por vía accesoria oculta y que luego de la ablación exitosa de la misma, no volvió a presentar síncope.

## Abstract (en)

The tachycardia-bradycardia syndrome is the most frequent form of presentation of Sick sinus node syndrome and is commonly characterized by episodes of paroxysmal atrial fibrillation followed by significant pauses, especially in older adult patients. Other frequently associated tachyarrhythmias are atrial tachycardia and atrial flutter. The association between orthodromic tachycardia and significant pauses in these patients is an unusual presentation. We present the case of an older adult with bradycardia-tachycardia syndrome and syncope, who presented with a hidden accessory pathway and who, after successful ablation of it, did not present syncope again.

## Introducción

El término «síndrome bradicardia-taquicardia alternante» fue acuñado en 1954 por Short, quien encontró en cuatro pacientes episodios de síncope debidos a bradicardia sinusal, los cuales ocurrían luego de episodios prolongados de taquicardia auricular ^1^. El tratamiento de esta entidad ha sufrido cambios a lo largo del tiempo, pasando por cirugías como tiroidectomía radical hasta el implante de marcapasos con resultados no siempre favorables, tal y como se muestra en reportes donde luego del implante de marcapasos los síntomas persisten y en la interrogación del dispositivo se evidencia escaso porcentaje de estimulación con episodios frecuentes de taquicardias supraventriculares ^2^^,^^3^. La ablación con radiofrecuencia de la taquiarritmia asociada se ha impuesto como el tratamiento de primera línea en la mayoría de los casos de síndrome taquicardia-bradicardia y ha demostrado ser una técnica eficaz ^3^.

## Reporte de caso

**Figura 1:**
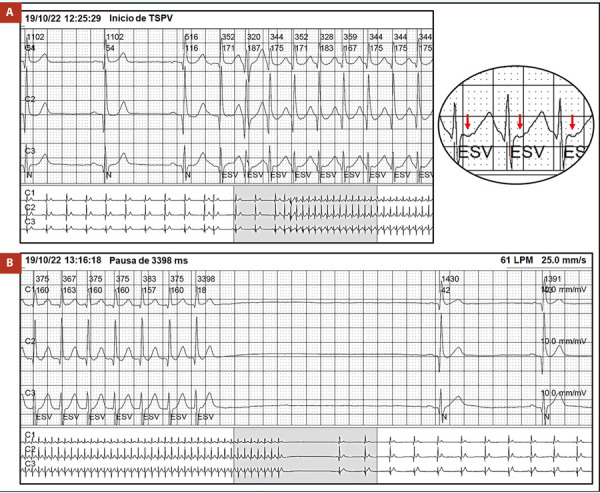
A) Inicio de taquicardia paroxística supraventricular con RR regular con una frecuencia de 170 latidos por minuto e intervalo RP mayor de 90 ms, las flechas rojas señalan las ondas P retrógradas. B) Pausa sinusal de 3,4 Segundos luego de un episodio de TPSV.

Varón de 74 años sin antecedentes cardiovasculares de importancia, con historia de síncope precedido de palpitaciones de dos meses de evolución, acude a un establecimiento de salud y le realizan una prueba Holter de 24 h donde se evidencia varios episodios de taquicardia paroxística supraventricular seguidas de pausas de hasta 3,4 s **(**Figura 1**)**. El paciente refiere haber presentado mareos y presíncope durante los episodios de taquicardia. Por ello fue referido a nuestro hospital para posibilidad y valoración de implante de marcapaso definitivo. El electrocardiograma basal tenía ritmo sinusal con frecuencia cardiaca alrededor de 88 latidos/min sin otras alteraciones significativas. El ecocardiograma transtorácico reveló función sistólica del ventrículo izquierdo normal sin valvulopatías y ausencia de cardiopatía estructural.

**Figura 2:**
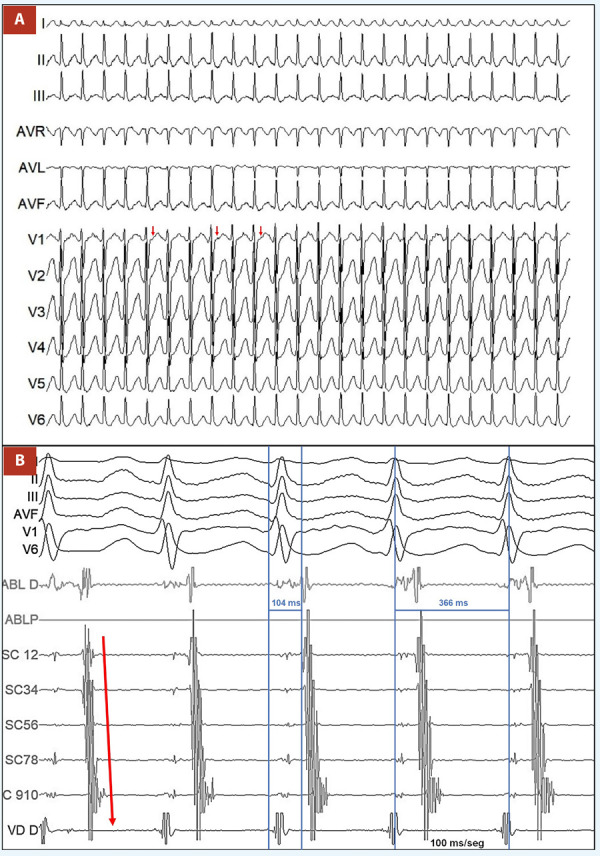
A) Taquicardia clínica en 12 derivadas con QRS estrecho, RR regular, RP < PR y onda P positiva en derivada v1. B) Registro intracavitario de la taquicardia clínica con longitud de ciclo de 366 ms, intervalo VA de 104 ms y activación auricular retrógrada excéntrica de distal a proximal (flecha roja). (SC: seno coronario, ABL: ablación, VD: ventrículo derecho).

**Figura 3:**
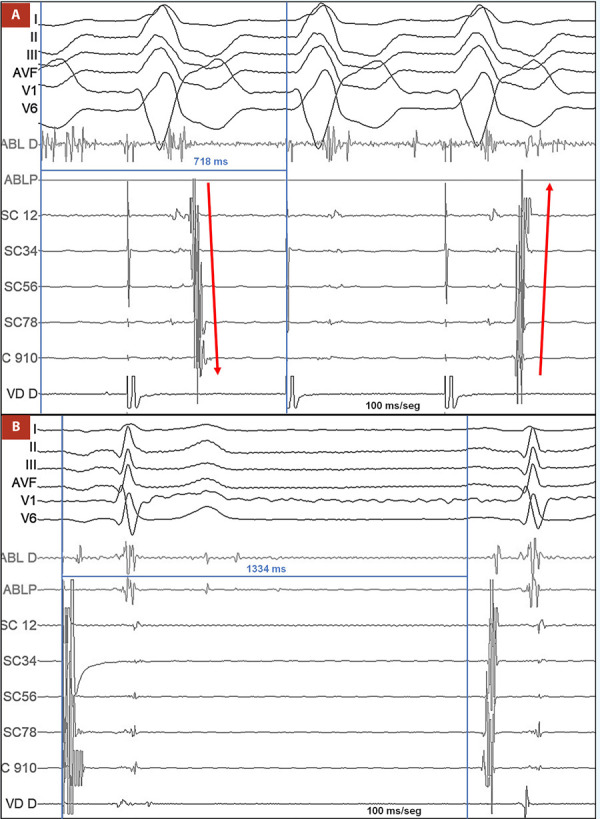
A) Aplicación de radiofrecuencia durante estimulación en el ventrículo derecho. Se evidencia pérdida de conducción a través de la vía accesoria a los 0,7 segundos. Se aprecia el cambio de activación auricular de un patrón excéntrico a un patrón concéntrico (flechas rojas). B) Tiempo de recuperación del nodo sinusal de 1334 ms tras estimulación auricular programada a 500 ms. Se midió desde la última onda P estimulada hasta el inicio de siguiente onda P.

Durante su hospitalización presentó múltiples episodios de palpitaciones y síncope asociados a la taquicardia, de carácter incesante que respondieron a adenosina endovenosa y maniobras vagales, por ello se decidió llevar a cabo estudio electrofisiológico, colocando un catéter decapolar en el seno coronario (SC) y un catéter cuadripolar en el ventrículo derecho (VD), durante el estudio se indujo la taquicardia clínica la cual era regular con QRS estrecho **(**Figura 2A**)**, longitud de ciclo de 366 ms y con activación auricular retrógrada excéntrica e intervalo VA de 104 ms, compatible con taquicardia ortodrómica por vía accesoria oculta izquierda **(**Figura 2B**)**, es por ello que se procedió a puncionar la arteria femoral derecha y por abordaje retroaórtico se avanzó un catéter de ablación irrigado (ABL) hacia el anillo mitral. Con el mencionado catéter se procedió a mapear la activación auricular retrógrada durante estimulación ventricular derecha. Durante el mapeo se obtuvo actividad auricular más precoz con VA fusionado y posible potencial de vía a nivel de la región ánterolateral del anillo mitral, lugar donde se aplicó radiofrecuencia (RF) controlada por poder (35 W) logrando interrumpir la conducción a través de la vía accesoria a los 0,7 segundos **(**Figura 3A**)**. Tras esperar 30 min de aplicada la radiofrecuencia y realizar maniobras de estimulación, la taquicardia no se volvió a inducir, por lo que la ablación fue catalogada como exitosa. Durante el procedimiento también se procedió a realizar pruebas de función sinusal de forma convencional ^4^, el tiempo de recuperación del nodo sinusal tras estimulación auricular programada fue de 1334 ms, el cual se encontraba dentro de límites normales (valor normal <1500 ms) **(**Figura 3B**).** En el seguimiento al año, el paciente no ha vuelto a presentar ningún episodio sincopal ni palpitaciones, y en los controles con Holter de 24 h no se ha encontrado episodios de taquicardia.

## Discusión

La ablación exitosa de taquiarritmias como fibrilación auricular en el contexto de síndrome taquicardia-bradicardia ha demostrado tener beneficios que, en determinados casos, superan al implante de un marcapaso, tales como mantener al paciente más tiempo en ritmo sinusal, evitar la progresión a la insuficiencia cardiaca e incluso de la misma arritmia ^5^. Otros tipos de taquicardia como la taquicardia por reentrada del nodo auriculo ventricular y la taquicardia ortodrómica mediada por una vía accesoria oculta seguida de pausas significativas, en adultos mayores, constituyen una forma de presentación poco frecuente del síndrome taquicardia-bradicardia ^6^. En este contexto la ablación con catéter por radiofrecuencia de la taquicardia ha demostrado ser un procedimiento que evita el implante de dispositivos de estimulación cardiaca ^3^^,^^7^^,^^8^.

Se asume que la disfunción del nodo sinusal en pacientes con síndrome taquicardia bradicardia se produce por un mecanismo denominado supresión por sobreestimulación; por tanto, dicha disfunción es secundaria a la taquiarritmia. En consecuencia, es razonable tratar primero la taquiarritmia antes de pensar en el implante de un marcapaso ^3^^,^^7^. Estudios recientes han demostrado la recuperación completa de la función del nodo sinusal luego de la ablación exitosa de la taquiarritmia primaria ^7^^,^^8^; además, en una serie de 51 pacientes con FA y pausas significativas, que fueron llevados a ablación, ninguno requirió marcapasos en el seguimiento ^8^. 

El presente caso ilustra una forma inusual de síndrome taquicardia-bradicardia, pues se trata de un paciente adulto mayor de 74 años, con historia de síncope a repetición debido a episodios de taquicardia ortodrómica seguidas de pausas significativas, gracias a que se realizó el estudio electrofisiológico y la ablación exitosa de la vía accesoria oculta se evitó exponerlo a los riesgos que un implante de marcapaso acarrea; así mismo, el tiempo de recuperación del nodo sinusal tras estimulación auricular programada fue normal, lo cual descarta de manera objetiva que el paciente tenga necesidad de marcapasos. Cabe señalar también que, en este caso en particular, al ser la taquicardia ortodrómica una macroreentrada que involucra tanto aurículas como ventrículos la estimulación del marcapasos no iba a evitar los episodios de taquicardia, por el contrario, la estimulación ventricular por el marcapasos hubiera hecho que la arritmia sea incesante ^2^^,^^3^.

## Fuente de financiamiento:

Autofinanciado
